# IMPLEMENTING THE LOOK AHEAD LIFESTYLE INTERVENTION IN PRACTICE AND COMMUNITIES: A SCOPING REVIEW

**DOI:** 10.1093/geroni/igae098.4004

**Published:** 2024-12-31

**Authors:** Yan Du, Lina Paola Buitrago Ubaque, Valeria Medrano, Erin Finley, Helen Hazuda, Sara Espinoza, Lixin Song, Elena Volpi

**Affiliations:** UT Health San Antonio, San Antonio, Texas, United States; University of Miami, Miami, Florida, United States; UT Health SA, San Antonio, Texas, United States; UT Health SA, San Antonio, Texas, United States; UT Health SA, San Antonio, Texas, United States; Cedars-Sinai Medical Center, Los Angeles, California, United States; University of Texas Health Science Center at San Antonio, San Antonio, Texas, United States; University of Texas Health San Antonio, San Antonio, Texas, United States

## Abstract

Diabetes is a public health concern as the population aging rapidly. The Look AHEAD study demonstrated that a lifestyle intervention could improve glucose and lipid control, mental health, physical function, quality of life, and the need for diabetes medications. We conducted a scoping review to explore the implementation of the Look AHEAD intervention in other populations and settings. We searched PubMed, Scopus and CINAHL in December 2023. Two researchers screened each article independently. The data extracted from the included studies were systematically tabulated and analyzed to identify study characteristics, key themes, trends, and gaps. The review included 21 articles from 8 study projects. Twelve articles reported on study methods or qualitative findings. Seven projects focused on adults (RCT), and one in older adults aged 65 and over (one arm, pre-and post- test). Interventions were delivered in clinical (4 projects), community (3 projects), and academic (1 project) settings, with sample sizes ranging from 20 to 331 participants. The number of educational sessions ranged from 10 to 60. Most studies incorporated self-monitoring, goal setting, psychological strategies, and social support for physical activity and diet. Three projects used meal replacements. Weight improved significantly in all intervention groups except one pilot trial. HbA1c improved across all groups, with only one study reporting significant results. Two studies assessed reduced diabetes medication use. The studies varied in sample size, intervention sessions, and settings. Overall, adapting Look AHEAD to other settings is feasible, but the effectiveness, adoption, and sustainability of implementing the adapted interventions warrant further investigation.

